# BARRIERS AND FACILITATORS OF DISASTER PREPAREDNESS FOR CAREGIVERS OF PERSONS WITH DEMENTIA

**DOI:** 10.1093/geroni/igac059.631

**Published:** 2022-12-20

**Authors:** Lindsay Peterson, Debra Dobbs, Sara Hackett, William Haley

**Affiliations:** University of South Florida, Tampa, Florida, United States; University of South Florida, Tampa, Florida, United States; University of South Florida, Tampa, Florida, United States; University of South Florida, Tampa, Florida, United States

## Abstract

Emergency preparedness for disasters such as hurricanes is critical. A common feature of disasters is their disruption of daily life, which is magnified for persons living with Alzheimer’s disease and related dementias (ADRD). It is critically important to understand more about disaster preparedness for those with ADRD and the informal caregivers responsible for their safety. We conducted individual interviews between April and September, 2021. The sample included 50 family caregivers of persons with dementia (11 Hispanic, 12 Black, 27 White), who were asked about their disaster experiences, concerns about future disasters and level of preparedness. Interviews were transcribed and coded using a team coding approach. Initial analysis identified three main themes, 1) caregivers attitudes about the importance of disaster preparedness, 2) what makes it difficult to prepare, and 3) facilitators of preparedness. Results have the potential to guide program development to improve preparedness among diverse caregivers of persons with ADRD.

